# Removal of fiber posts using conventional versus guided endodontics: a comparative study of dentin loss and complications

**DOI:** 10.1007/s00784-024-05577-7

**Published:** 2024-03-04

**Authors:** R. Krug, F. Schwarz, C. Dullin, W. Leontiev, T. Connert, G. Krastl, F. Haupt

**Affiliations:** 1 Department of Conservative Dentistry and Periodontology and Center of Dental Traumatology, University Hospital of Würzburg, Pleicherwall 2, 97070 Würzburg, Germany; 2 Department for Preventive Dentistry, Periodontology and Cariology, University Medical Center Göttingen, Göttingen, Germany; 3 Department of Periodontology, Endodontology and Cariology, University Center for Dental Medicine, University of Basel, Basel, Switzerland

**Keywords:** Endodontic retreatment, Fiber post removal, Guided endodontics, Dentin loss, Procedural error

## Abstract

**Objectives:**

To compare the efficacy of fiber post removal using conventional (CONV) versus guided endodontics (GE) in terms of dentin loss, residual resin material, procedural errors, and working time in vitro.

**Material and methods:**

Ninety human central incisors were root-filled and scanned by micro-computed tomography (CT), then restored with fiber posts and composite. Twenty-four sets of teeth with up to four human maxillary central incisors were fabricated and divided into three groups: conventional post removal by a general dentist (CG) or endodontology specialist (CS) and guided endodontics (GE) by a general dentist, yielding 30 teeth per operator and group. After treatment, the prepared access cavities were volumetrically assessed by micro-CT. Statistical significance was evaluated by one-way analysis of variance followed by post hoc comparisons with Tukey's HSD test and Pearson's chi-squared test for independence.

**Results:**

Both CONV and GE resulted in dentin loss and residual resin material. CS resulted in more dentin loss and less residual resin material than CG and GE (p < .05). All groups had some deviations from the original root canal but no perforations. The shortest working time was observed in the GE group.

**Conclusions:**

Compared to the conventional freehand technique, GE resulted in significantly less radicular dentin loss, a few deviations but no perforations.

**Clinical relevance:**

Guided endodontics can improve the speed and safety of fiber post removal without root perforation.

## Introduction

Endodontic retreatment of root-filled teeth with periapical lesions can be time consuming and challenging. The goals are to gain access to the endodontic system to remove the entire root canal filling, perform chemo-mechanical disinfection, and promote apical healing [1, 2]. If present, intraradicular posts must be carefully removed to access the root canal filling in the apical part of the root. When removing the posts, it is important to preserve as much radicular dentin as possible and avoid procedural errors such as deviation from the original root canal (via falsa) or perforation. The high risk of iatrogenic damage due to post removal is well known [3]. Adequate radiographic diagnosis, a high level of clinical experience of the operator, and in cases of fractured posts the use of (ultra-)sonic instruments [4] appear to be mandatory for safe and effective post removal [5, 6].

There are several types of posts, which may be made of alloys or ceramics or be fiber-reinforced with composite resin material. The purpose of all post systems is to restore root-filled teeth with severe hard tissue loss [7]. The upward trend in restoring such teeth with fiber posts may be related to their tooth-colored appearance and dentine-like mechanical properties, which are considered to be more biomimetic [8]. Although fiber posts are less fracture resistant than metal posts, failed fiber posts are usually restorable [9]. The ferrule effect is another important factor to consider when restoring severely damaged root-filled teeth. In a systematic review, the ferrule effect was shown to be more important than post type with regard to tooth and restoration survival after endodontic treatment [7, 10]. Fiber posts must be adhesively bonded to the root canal dentin and the remaining root canal space must be filled with luting material. However, adhesive luting can impede the removal of fiber posts, increasing the risk of procedural errors, especially when attempting to debond posts located in deep root canal spaces with poor access and visibility. To improve safety, several researchers have investigated the dentin loss and residual composite associated with different post removal techniques. High levels of radicular dentin loss could weaken the teeth and leading to dentinal microcracking – one of many effects that have been discussed as potential causes of vertical root fractures in root-filled teeth [11]. Interestingly, a recent in vitro study comparing the effectiveness of three different fiber post removal techniques found no correlation between dentin loss and the induction of microcracks [3].

Clinically, the use of guided endodontics (GE) with an endodontic access drill has been shown to provide straight-line access to the root canal system of teeth with pulp canal obliteration (PCO) and periapical radiolucency [12–14]. GE is often used to treat more difficult cases such as those with PCO [15]. However, it is technically challenging in that tooth surface scan data and cone beam computed tomography (CBCT) data must be matched using special planning software to virtually visualize the target root canal. Once the position of the drill path has been defined, a drill guide with a sleeve must be designed and fabricated.

Another advantage of guided endodontics is that it facilitates root canal location in teeth with calcified pulp chambers due to dentin dysplasia, a rare developmental disorder [16]. In addition, guided endodontics allows minimally invasive access to the apices during endodontic surgery [17–19]. A few case reports have shown that guided endodontics can be used to successfully remove fiber posts from root-filled teeth [20–26]. The accuracy of GE to provide access for the removal of intraradicular fiber posts was evaluated in an in vitro study of 40 teeth restored with fiber posts and composite cores, which showed that GE resulted in a mean apical deviation of 0.40 ± 0.19 mm [27]. However, to be successful, fiber posts must be removed in a time-efficient and safe manner without unnecessary loss of dentin or procedural errors. To our knowledge, this is the first comparative study using micro-CT for volumetric quantification of residual resin material after fiber post removal.

The aim of the present study was to evaluate the effectiveness of two methods of fiber post removal (conventional versus guided endodontics) in terms of dentin loss, residual resin material and working time when performed by an endodontology specialist and/or a general dentist. Procedural errors were additionally recorded. The null hypothesis was stated that there is are no differences between the post removal methods (conventional and guided endodontics) in terms of effectiveness and the occurrence of procedural errors.

## Materials and methods

Ninety human maxillary central incisors were selected according to the following criteria: complete tooth crown without extensive carious lesions or restorations, straight mature root with an untreated root canal, length between 20 and 24 mm. All teeth were stored in 1.0% chloramine T solution, which has been reported to be an appropriate storage medium with no relevant effect on dentin bond strength [28].

Root canal preparation was performed with reciprocating nickel-titanium instruments (Reciproc R50; VDW Dental, Munich, Germany) according to the manufacturer's instructions. During preparation, the root canals were irrigated with 3% sodium hypochlorite (total volume: 3 ml). All posts (D.T. Light-Post size 3, VDW Dental) were shortened to a standardized length of 10 mm. Root canals were obturated using a single-cone technique with gutta-percha and sealer (AH plus, Dentsply Sirona, Ballaigues, Switzerland) with warm vertical compaction. Depending on the tooth length, the obturation lengths varied to meet the goal of placing each post 7 mm apical to the cementoenamel junction. The accuracy of the measured distances was assessed clinically using an endodontic measuring gauge and radiographically using the Sidexis 4.3 measuring tool (Dentsply Sirona).

Root canals were then prepared with the D.T. Light-Post Finishing Drill #3 (VDW Dental) and cleaned with ethyl alcohol (80%). All teeth were scanned preoperatively with a micro-CT scanner (QuantumFX, Perkin Elmer, Waltham, MA, USA) using the following settings: tube voltage 90 kV, tube current 200 µA, field of view 20 × 20 mm, total acquisition time two minutes. This resulted in volumetric data sets with a matrix size of 512 × 512 x 512 voxels and a reconstructed isotropic voxel size of 39 µm. Preoperative access cavity volumes were estimated from the binarized images using CTan v.1.20.3.0 software (Bruker-μCT, Kontich, Belgium).

Prior to post insertion, all root canals were conditioned with 36% phosphoric acid for 15 s (s), rinsed with distilled water for 20 s, and dried with paper points. A mixture of Prime&Bond XP (Dentsply Sirona) and Self-cure Activator (1:1) was applied to the root canal walls and posts for 20 s. Root canals were filled with Core-X flow (Dentsply Sirona) and posts were immediately placed to the full depth of 10 mm. Adhesive sealing of the coronal end of the post was ensured and light polymerization was applied for 40 s.

Twenty-four maxillary models with up to four human maxillary central incisors each were fabricated as part of an upper jaw set. The extracted teeth were removably inserted into a resin mold and each dental arch was completed with 3D printed teeth. The 24 models were divided into three groups according to post removal technique and operator as follows: conventional endodontics by an endodontology specialist (n = 8) or general dentist (n = 8) and guided endodontics by a general dentist (n = 8).

**Fig. 1:**
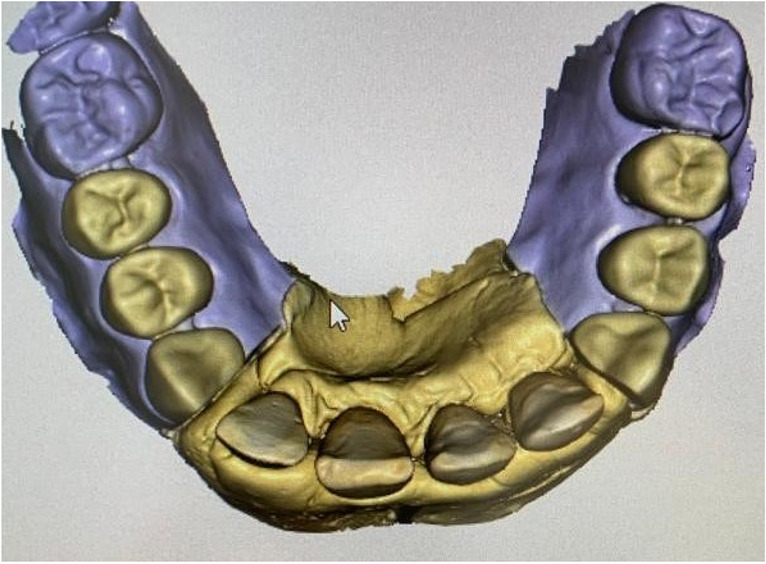
Surface scan of a maxillary model with four human central incisors in a resin mold adjacent to 3D printed replicas of the canines and premolars

**Fig. 2:**
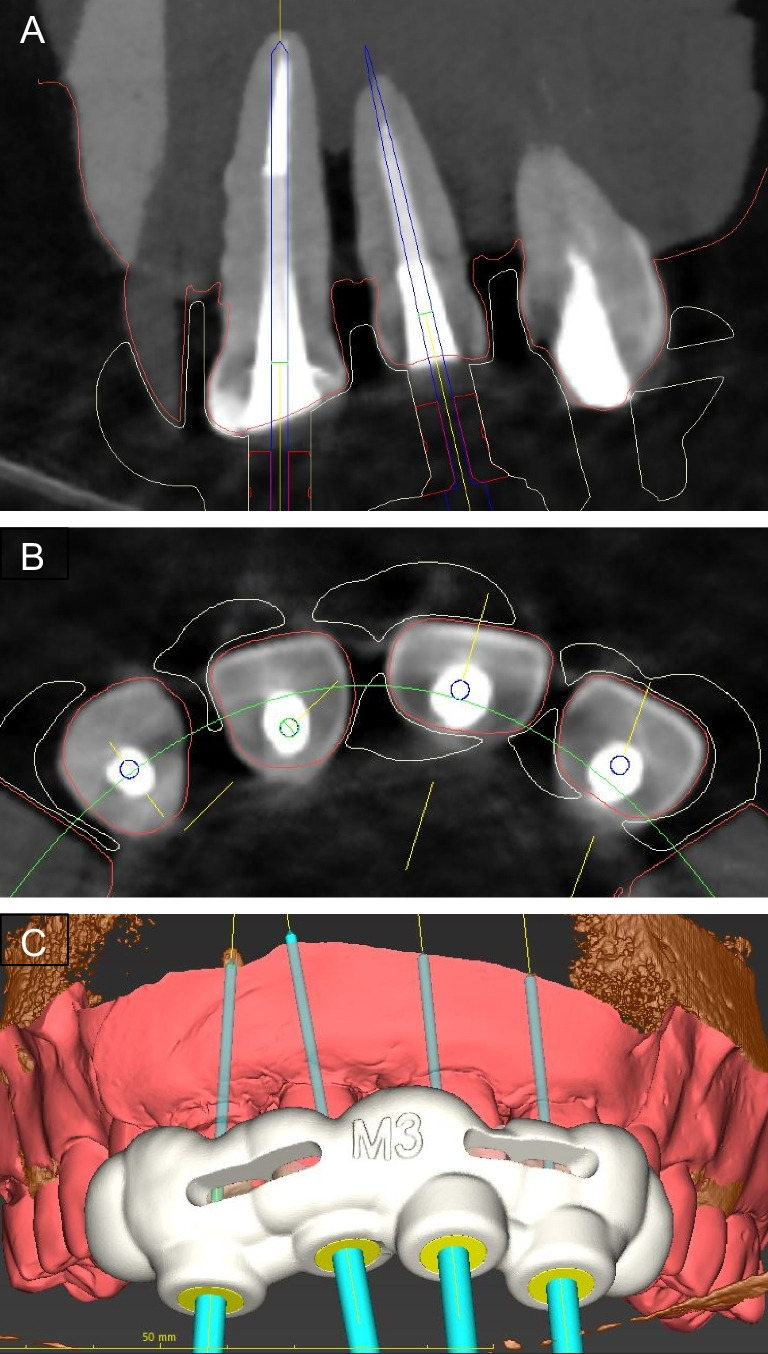
The guided endodontics technique with a virtually planned drill path targeting the root canal filling: coronal view (**A**), axial (**B**) view, and 3D rendered view of the matched surface scan and CBCT data with four planned access cavities and the designed template (**C**)

The data sets of the corresponding surface scans (Sirona CEREC Primescan AC, Dentsply Sirona) (Fig. 1) and CBCT scans (Orthophos SL 3D, Dentsply Sirona) were matched using implant planning software (coDiagnostiX 9.0, Dental Wings Inc., Chemnitz, Germany). Root canal access was planned by virtually placing a true-to-size drill toward the root canal [29, 30]. The tip of the drill was virtually aimed at the root canal filling. The drill path was always located in the center of the fiber post in three-dimensional space. After virtual access planning, a drilling template was designed and 3D-printed for each model (Objet 30 DentalPrime, Stratasys Inc., Rheinmünster, Germany). Metal sleeves (steco-system technik GmbH, Hamburg, Germany) were inserted into the fabricated drilling templates to guide the 1.0 mm drill (Endoseal, ATEC Dental Inc., Ebringen, Germany) (Fig. 2).

To simulate clinical conditions, the models were mounted in a dental patient simulator (KaVo Dental patient simulator, Kavo Dental Inc., Biberach an der Riß, Germany) that was fixed on the chair of a dental unit (KaVo Esthetica, Kavo Dental Inc.). In the three treatment groups, conventional post removal was performed by both a general dentist (CG) and an endodontology specialist (CS), while guided endodontic post removal was performed by a general dentist alone (GE). Conventional post removal was carried out using long-shank bud burs (diameter: 1 mm, two teeth per bur). In all groups, SonicFlex Endo tips were used under a dental operating microscope (12.5X magnification; Zeiss Pico, Zeiss, Jena, Germany) removing residual material of post or resin. The times required to reach the coronal end of the root canal filling and to remove the post were recorded. Complete removal of the resin material was verified under a dental operating microscope in all groups.

**Fig. 3:**
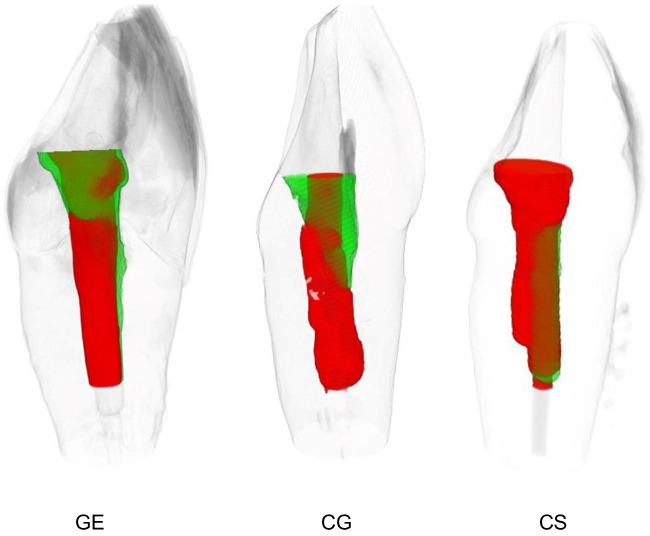
Representative samples showing dentin loss (red) and residual resin material (green) after fiber post removal in the three different groups: guided endodontics by a general dentist (GE), conventional endodontics (freehand technique) by a general dentist (CG), and conventional endodontics by an endodontology specialist (CS)

Post-treatment micro-CT scans were obtained using the initial parameter settings. Pre and post data sets were co-registered in DataViewer software (v. 1.5.6.1, Bruker-μCT, Kontich, Belgium) using a pseudo-3D registration tool. CTan v.1.20.3.0 software (Bruker-μCT) was used to calculate quantitative variables. For volumetric analysis, dentin loss and residual resin volume were calculated by subtraction (pre minus post). In addition, procedural errors such as perforation and deviation from the original root canal without perforation were evaluated (Fig. 3).

Statistical analysis was performed with SPSS (Version 28.0.1.1, IBM Corp., Armonk, USA) using one-way analysis of variance (ANOVA) followed by post hoc comparisons with Tukey’s HSD test and the Pearson’s chi-squared test for independence. The level of statistical significance was set at α = 0.05.

## Results

**Fig. 4:**
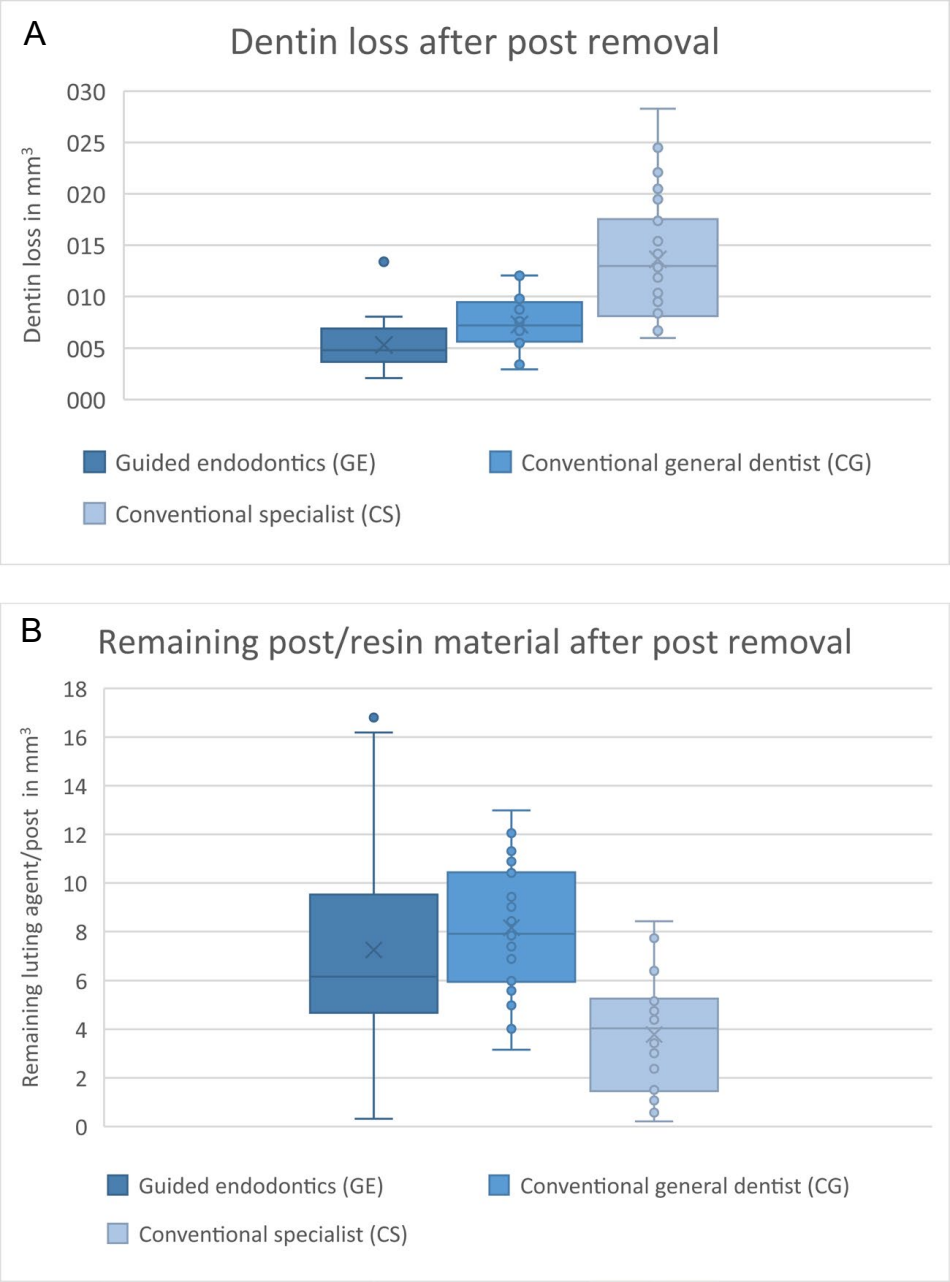
Distribution of dentin loss (**A**) and residual resin/fiberglass (**B**) in the three groups

Both post removal techniques resulted in dentin loss and residual resin material. Conventional post removal by an experienced specialist (CS) resulted in significantly more dentin loss and less residual material compared to CG and GE (Table 1, Fig. 4). ANOVA revealed a significant difference in dentin loss (F [2, 48,862] = 38,188, *p* < 0.001, η^2^ = 0.467), residual material (F [2, 68,097] = 16,134, *p* < 0.001, η^2^ = 0.271), and the time required to access the gutta-percha between the three groups (F [2, 67,284] = 14,060, *p* < 0.001, η^2^ = 0.244), but no significant difference between the three groups in the time required to detect a free dentinal wall around the fiber post or luting agent, as verified by dental microscopy (F [2, 44,165] = 1.665, *p* = 0.201).

**Table 1:** Dentin loss, residual resin/fiberglass, and working times in the three groups, expressed as mean values with standard deviation; GE: guided endodontic post removal by a general dentist, CG: conventional post removal by a general dentist; CS: conventional post removal by a specialist

	Dentin loss	Residual resin and	Working time	Working time
Treatment	after post	fiberglass after	Time to access	Cavity preparation with complete
group	removal [mm^3^]	post removal [mm^3^]	gutta-percha [min]	post /resin removal [min]
GE	5.32 ± 2.26^a^	7.24 ± 4.16^a^	3.05 ± 1.68^a^	5.88 ± 1.10^a^
CG	7.29 ± 2.44^a^	8.16 ± 2.54^a^	6.06 ± 1.82^b^	7.01 ± 1.74^a^
CS	13.62 ± 5.76^b^	6.40 ± 3.64^b^	4.96 ± 2.53^b^	6.87 ± 4.04^a^

Deviation from the original canal without perforation occurred in all three groups (Table 2). Pearson’s chi-squared test for independence revealed no significant difference in the number of deviations between groups (χ^2^[2, n = 90] = 4,845, *p* = 0.089). No perforations occurred. The shortest working time was observed in the GE group (Tables 1 and 2). Pearson’s chi-squared test for independence revealed a significant difference in the number of perforations between groups (χ^2^[2, n = 90] = 6,3, *p* = 0.043, Cramer's V = 0.265).

**Table 2:** Procedural errors: deviation from the original GE: guided endodontic post removal by a general dentist, CG: conventional post removal by a general dentist; CS: conventional post removal by a specialist canal without perforation (deviation) and perforation in the three groups

Treatment group	Deviation *n* (%)	Perforation *n* (%)	Cavities prepared without procedural error *n* (%)	Total *n* (%)
GE	4 (13.3)	-	26 (86.7)	30 (100)
CG	6 (20.0)	4 (13.3)	20 (66.7)	30 (100)
CS	11 (36.7)	6 (20.0)	13 (43.3)	30 (100)

## Discussion

Guided endodontics is reported to be an efficient and technically sophisticated method of locating calcified root canals in more difficult endodontic cases [15]. It is also used in other clinical applications, such as guided apical surgery [17] or guided drilling with a template for fiber post removal [27]. This is the first in vitro study to objectively demonstrate the benefits of guided endodontics to the operator in case of post removal. In the present study, the guided approach achieved three important advantages over the conventional technique, irrespective of operator experience: a mean 37% reduction of dentin loss, a mean 2 to 3-min reduction of time required to access the root canal filling, and the elimination of root perforation. The null-hypothesis had to be rejected. GE resulted in significantly less radicular dentin loss and no perforations compared to the conventional freehand technique.

Previous studies investigating the loss of dental hard tissue due to various post removal techniques have demonstrated the usefulness of both special fiber post removal kits and conventional burs, e.g., long-shank bud burs [3, 31]. Post removal, a common endodontic procedure, is the first challenge of orthograde retreatment before removing infected root canal fillings. The risk of root fracture during this procedure is considered to be very low when performed by an experienced operator [32]. In a clinical study of 1600 teeth in which posts were removed by an endodontology specialist and the tooth structure was examined by light transillumination immediately after post removal and during all subsequent endodontic retreatments, the incidence of root fracture was less than 1% [33]. Interestingly, the most common type of post was a cast post/core system, which had to be bypassed by removing the luting material, probably using ultrasonic vibration [33].

Modern fiber posts are notoriously difficult to remove. Due to the use of dentin-colored luting resin and core materials, it is often difficult to distinguish between these materials and dental hard tissues. In addition, because fiber posts, unlike metal posts, are adhesively bonded to the root dentin and must be completely removed by drilling, there is a higher risk of damaging sound radicular dentin. The related increase in dentin loss is associated with a decrease in post-endodontic tooth stability, which appears to be associated with a higher risk of dentinal crack formation. In the long term, vertical root fractures may occur. However, to date, there is no evidence of a direct relationship between dentin loss as a consequence of fiber post removal and the occurrence of microcracks [3]. The results of a narrative review suggest that dentinal microcrack formation is a unique phenomenon of laboratory studies, limited to extracted teeth, and caused by dehydration and in vitro storage conditions [34]. Iatrogenic damage to tooth structure is a known adverse event of fiber post removal in vivo [5]. In clinical conditions, the prognosis of cracked teeth (due to the presence of post misplacement, tooth or amalgam wear) seems to benefit from restorations with full-crown coverage after endodontic treatment [35].

In the present study, all fiber posts were placed in the apical third of root canals of uniform length according to a standardized protocol. The depth of post insertion makes it more difficult to remove fiber posts without damaging sound dental hard tissue. Conventional post removal, performed by a specialist with 15 years of endodontic experience using a dental microscope, resulted in three times more dentin loss than the guided endodontic method. In addition, the incidence of deviation from the original root canal without perforation was almost three times higher. Conventional treatment by a general dentist and a specialist resulted in a low rate of root perforation compared with no perforation in the guided endodontics group. These results indicate that the guided approach is a very safe and feasible technique that is not dependent on operator experience or skill level. Because a previous study of guided endodontics versus static navigation had already shown that guided endodontics allows more efficient location of simulated calcified root canals with significantly less substance loss, independent of operator experience [30], it was decided to have only one investigator perform the guided endodontic treatment in the present study.

Interestingly, fiber post removal by the specialist resulted in significantly more dentin loss than removal by a general dentist. The general dentist removed the post very cautiously, resulting in significantly more residual material. These discrepancies may be due to the fact that experienced endodontists generally strive to remove the entire post and resin material as meticulously and accurately as possible. However, in difficult cases, it may be more appropriate and prudent to leave some remnants of the post or luting material in situ to prevent further damage to the tooth structure. From a technical perspective, the clinician's goal should be to locate the microbially colonized gutta-percha in the apical part of the root without making any procedural errors. Clinically, one must weigh the pros and cons of the orthograde feasibility of complete root filling removal or the treatment option of retrograde surgery.

There were several methodical limitations of the present study. In all groups the operator could collate the clinically selected axis of the bur with the radiographically marked axis of the post on a periapical radiograph. To simulate a common clinical situation, the benefits of having an eccentric radiograph or using 3D imaging during post removal were not evaluated. Further, the endpoint of complete post removal was detected visually using magnifying spectacles and dental microscope. Skills, experience and intrinsic motivation of the operator might affect the results using visual magnification and detecting various residual materials on the dentin. Further, drilling onto a fibre post was associated with specific risks, e.g. rapid wear of the drill and heat development. Post removal was performed in up to two teeth using one drill (Endoseal or long-shank bud bur) with permanent water cooling. High qualities of the edges of each drill were made sure visually at all times. In general, it was focused on a high standardization of the methodology during the technical procedure of the post removal and the radiographic micro-CT imaging process. Technical efforts were made in order to reduce artefact formations due to the rotation of the samples during scanning process. The reproducibility of the tooth position was achieved by a 3D registration process for pre- and posttreatment micro-CT scans followed by a preliminary DataViewer-supported superposition and, finally, a visual vernier adjustment from one skilled radiologist.

In the present study, micro-CT was used for three-dimensional visualization and quantitative assessment of dentin loss and residual resin material. Other investigators have used CBCT to evaluate tooth substance loss or drill path accuracy during guided access cavity preparation using static and dynamic navigation systems [13, 30, 36, 37]. Three studies also used micro-CT to measure volumetric changes before and after post removal [3, 38, 39]. Currently, micro-CT represents the gold standard for the evaluation of volumetric changes in the root canal due to endodontic procedures as it allows 3D reconstruction of teeth and precise calculation of substance loss with high spatial resolution [34].

Future directions may include real-time 3D motion tracking for fiber post removal under difficult conditions, such as thin roots with a post inserted in the middle or apical third of the root canal. Dynamic navigation during fiber post removal not only allows for maximum preservation of dental hard tissue [40], but also increases the accuracy and efficiency of post removal compared with conventional techniques [41]. It also reduced the time required for post removal from a mean of 8.30 ± 4.65 min with conventional methods to only 4.03 ± 0.43 min. However, 3D navigation requires enormous technological effort and resources [41]. So far, case reports and preliminary in-vitro studies indicate that dynamic navigation is an ultramodern technique to manage challenging clinical situations, e.g. pulp canal obliteration, (ultra-)conservative access preparation, retreatment or microsurgery with fewer procedural errors in a shorter time among operators with all levels of experience [42–46].

Interestingly, in the present study, the mean time for complete post and resin removal was only 5.88 ± 1.10 min in the static navigation group and 6.87 ± 4.04 min and 7.01 ± 1.74 min in the freehand navigation groups. In comparison, navigation only slightly reduced the working times for post removal, so the time reduction aspect does not appear to be the most relevant argument for using either static or dynamic navigation techniques in clinical practice. As an alternative to the conventional procedure used in the present study, time-efficient and complete fiber post removal can also be achieved with a special removal kit described previously [47]. However, to our knowledge, these fiber post removal kits are no longer commercially available. The promising results of the present study, with its strengths and limitations, underscore the clinical relevance of static navigation for guided fiber post removal in endodontics.

## Conclusion

In this study, the novel technique of guided endodontics significantly increased the time efficiency and safety of fiber post removal, particularly from the apical third of the root canal, decreasing the mean working time to approximately six minutes. Guided endodontics significantly reduced radicular dentin substance loss compared with conventional endodontics, regardless of whether performed by a general dentist or an endodontology specialist. In addition, no perforation and only a few deviations from the original root canal without perforation were observed with guided endodontics.

## Data Availability

No datasets were generated or analysed during the current study.
